# Validation of the Leicester Cough Questionnaire in Brazilian Portuguese

**DOI:** 10.1590/2317-1782/e20240391en

**Published:** 2025-09-04

**Authors:** Rodrigo Dornelas, Vanessa Veis Ribeiro, Alice Lopes, Thassiany Carpanez, Surinder Birring, Mara Behlau

**Affiliations:** 1 Departamento de Fonoaudiologia, Universidade Federal do Rio de Janeiro – UFRJ - Rio de Janeiro (RJ), Brasil.; 2 Departamento de Fonoaudiologia, Universidade de Brasília – UnB - Brasilia (DF), Brasil.; 3 Centre for Human and Applied Physiological Sciences, King’s College London - London, United Kingdom.; 4 Centro de Estudos da Voz - São Paulo (SP), Brasil.

**Keywords:** Cough, Chronic Cough, Validation Study, Self-testing, Disease, Larynx

## Abstract

**Purpose:**

This study aimed to validate the Leicester Cough Questionnaire (LCQ) for Brazilian Portuguese.

**Methods:**

Validation followed the Consensus-Based Standards for the Selection of Health Measurement Instruments (COSMIN). Data collection included a sociodemographic questionnaire, the translated version of LCQ-Brazil (LCQ-Br), self-perception of laryngeal sensitivity, cough frequency and intensity, the Cough Severity Index (CSI-Br), and the Newcastle Laryngeal Hypersensitivity Questionnaire (LHQ-Br). The LCQ-Br retained its original structure with 19 items across physical, psychological, and social domains. Participants completed the LCQ-Br on three occasions to assess validity, reliability, and responsiveness.

**Results:**

Ninety-eight patients with chronic cough (79% women; mean age of 49) participated. Construct validation confirmed the LCQ-Br's factorial structure. For concurrent validity, negative correlations were observed between LCQ-Br domains and self-perceived laryngeal sensitivity, cough frequency and intensity, and CSI-Br factors (physical, social, psychological, and total scores). A positive correlation was found between the LCQ-Br total and LHQ-Br scores. Internal consistency was high (Cronbach's alpha = 0.952), and test-retest reliability yielded a coefficient of 0.455. The responsiveness analysis demonstrated significant reductions in LCQ-Br scores post-intervention for physical, psychological, and total domains.

**Conclusion:**

The LCQ-Br is a valid, reliable, and responsive tool for assessing health status in chronic cough patients, making it suitable for clinical practice and research applications.

## INTRODUCTION

In respiratory physiology, the cough reflex plays an important role in protecting the lower airways, preventing the aspiration of unwanted materials into the lungs. This defense mechanism is regulated by a complex reflex arc that is activated by the stimulation of specific sensory receptors located in the larynx and upper airways^(1)^. However, in some cases, coughing is not considered beneficial and must be controlled. In the United States, cough is the most common complaint during health consultations and represents approximately 30 million visits per year^(2)^. In England, the prevalence of cough corresponds to 12% of medical complaints^(3)^; in Brazil, it can reach 11.6%^(4)^.

When cough persists manifestation for more than 8 weeks, it is categorized as chronic. Chronic cough (CC) is a multifaceted symptom that challenges clinical understanding, requires varied approaches, and can significantly affect patients’ quality of life^(5)^.

In cases of CC, speech therapy has emerged as a potential treatment option. Despite being a relatively recent area of research, there is already evidence supporting the effectiveness of speech therapy for CC^(6-8)^. For successful speech therapy, it is essential to perform a specific evaluation of this condition. Understanding the functional, social, and psychological implications of persistent cough forms the foundation for designing unique therapeutic plans.

Among the Speech-Language Pathology (SLP) clinical evaluation procedures for CC, self-assessment of this symptom plays a crucial role. One of the most commonly used instruments for the self-assessment of CC is the Leicester Cough Questionnaire (LCQ) (Leicester Questionnaire on Chronic Cough - LCQ-Br).

In the PubMed research platform database, more than 350 references have used LCQ, which shows the wide acceptance and applicability of this instrument in clinical practice and studies related to CC. This extensive use of the LCQ highlights the importance of reliable and validated tools for evaluating the impact of CC on patients’ quality of life. The LCQ has been shown to be the preferred choice among health professionals for this purpose, justifying the need for validation in different linguistic and cultural contexts^(9)^.

The original version of the LCQ, developed in English by Birring et al.^(10)^, stands out as a comprehensive measure that assesses cough frequency, cough intensity, and its impact on quality of life. Its application in a language different from the original requires rigorous validation to ensure the necessary sensitivity for capturing the peculiarities of CC, in our case, considering the Brazilian population.

The LCQ-Br has already been translated and adapted for Brazilian Portuguese (BP)^(11)^, however, it has not yet been fully validated. The validation process can identify and modify aspects of the instrument that may not be applicable or interpretable in BP in the same way as the original, due to sociocultural differences. This validation process will contribute to the development of a sensitive and specific instrument for the evaluation of CC, ensuring its applicability and accuracy in the Brazilian context and facilitate the proper identification and management of CC^(5)^. Thus, we sought to provide another resource for the evaluation of CC in Brazil to support the effective management of this condition.

The inclusion of a new, validated evaluation instrument represents an advancement for speech therapists and other health professionals in the management of patients with CC. This instrument will improve the ability of professionals to identify the impact of CC and also to intervene effectively in cases of CC, thereby contributing to the well-being and quality of life of individuals with this condition^(12)^.

Thus, this study aims to validate the Leicester Cough Questionnaire in Brazilian Portuguese.

## METHODS

This cross-sectional study was approved by the Research Ethics Committee of the Universidade Federal do Rio de Janeiro (Protocol 4.789.449). All ethical precepts were complied with in accordance with Brazilian legislation.

The study was conducted based on the version of the instrument previously translated and adapted into Brazilian Portuguese.

The validation procedures followed the standards defined by the Consensus-based Standards for the Selection of Health Measurement Instruments (COSMIN)^(13,14)^.

The calculation of the sample size was based on data from a pilot study using the measurements of the first five participants of the experimental group, referring to the Newcastle Laryngeal Hypersensitivity Questionnaire - LHQ-Br^(15)^, a self-assessment questionnaire^(16)^, administered before and after the intervention, to estimate the variability of the data. Variability was quantified as the standard deviation of the difference between the pre- and post-intervention scores, which was 10.4, with this magnitude considered the minimum relevant change observed in the total score. With significance level established at 5% and a statistical power of 90% to identify a minimum change of 10.4, the minimum number of participants required for each study group was calculated as 12.

The recruitment of research participants was carried out at the Hospital Universitário Clementino Fraga Filho of Universidade Federal do Rio de Janeiro in the Pulmonology and Otorhinolaryngology outpatient clinics. The participants were diagnosed with CC and provided informed consent by signing an Informed Consent Form (ICF). The data were collected at a Speech Therapy Outpatient Clinic.

The inclusion criteria were being 18 years or older, having a medical diagnosis of CC, and having BP as their native language. Participants who did not agree to participate in the study by signing the FICF were excluded from the study.

Data collection included a sociodemographic questionnaire, LCQ-Br, self-perception of laryngeal sensitivity, cough frequency, cough intensity, the Cough Severity Index - CSI-Br^(17)^ and the LHQ-Br^(15)^. The sociodemographic questionnaire was developed by the authors and included data on age, gender, nationality, and CC diagnosis.

To analyze the self-perception of laryngeal sensitivity, cough frequency, and cough intensity, a 10 cm visual analog scale (VAS) was used, in which the participant was instructed to record how they perceived the aforementioned aspects. At the extremities of the VAS, the responses varied between “Adequate” and “Very Sensitive” for laryngeal sensitivity, “Never” and “Always” for cough frequency, and “Adequate” and “Extremely Strong” for cough intensity.

The translated and cross-culturally adapted version of the LHQ-Br^(15)^ maintained its original structure, with 19 items organized into three domains: physical (items 1, 2, 3, 9, 10, 11, 14, and 15), psychological (items 4, 5, 6, 12, 13, 16, and 17), and social (items 7, 8, 18, and 19). The responses were quantified by the respondents on a Likert scale ranging from 1 to 7 points and referred to the last two weeks. A score was obtained using the simple sum of the responses in each domain. The overall score is the sum of the individual scores for each domain^(10,11)^.

The CSI-Br consists of ten symptoms related to chronic cough (CC) in different contexts. The patient is instructed to respond to the frequency of these symptoms using a Likert scale ranging from 0 (never) to 4 (always). The higher the score, the greater the presence of symptoms^(17)^.

The participants responded to the LCQ-Br on three different occasions: Moment One, called the test; Moment Two, 14 days after Moment One, called the retest; and Moment Three, after the SLP intervention^(18)^. In addition, moment two also involved the self-perception of laryngeal sensitivity, cough frequency and intensity, and completion of the CSI-Br and LHQ-Br questionnaires.

The psychometric properties of the LCQ-Br included validity (construct and concurrent criterion validity), reliability (reliability and internal consistency), and responsiveness^(13)^.

The analysis considered a 95% confidence interval and a significance level of 5%. The statistical software IBM SPSS Statistics 29.0 and IBM SPSS Amos 29.0 were used.

The LCQ-Br (Appendix A) follows a theoretical structure of factors. Thus, for construct validation, confirmatory factor analysis (CFA) was used^(19)^. The CFA examined the factorial structure of the Leicester Cough Questionnaire and to confirm its construct validity. In the CFA, the latent factors were defined based on the theoretical structure of the original version. The following fit indices were measured: adjustment adequacy index (GFI), adjusted adjustment adequacy index (AGFI), standardized adjustment index (NFI), relative adjustment index (FRI), Parsimonious standardized adjustment index (PNFI), and standardized average quadratic residue (SRMR)^(20)^.

To verify concurrent criterion validity (i.e., the extent to which the questionnaire correlates with other measures based on the same theory and concept), the LCQ-Br was compared with self-perceived laryngeal sensitivity, cough frequency and intensity, CSI-Br^(17)^, and LHQ-Br^(16)^. Spearman’s correlation test was used.

To analyze internal consistency reliability, individuals with CC responded to the instruments once, and their responses were analyzed using Cronbach’s alpha coefficient.

Test-retest reliability was calculated to assess the stability of the instrument over time. Two evaluations were carried out at an interval of 14 days, as recommended to prevent significant changes in the evaluated domains^(21,22)^. The calculation was based on a comparison of the questionnaire results at test and retest moments. An intraclass correlation coefficient (ICC) was calculated.

To measure responsiveness, changes in instrument scores were analyzed in relation to changes in the measured construct. Individuals with CC responded to the instrument before and after eight therapy sessions, which were adapted to the individual needs of each participant. The objective was solely to verify whether the instrument could detect changes in the patient's condition resulting from rehabilitation for CC. The Wilcoxon test was used to compare the data from the instrument at two evaluation time points.

## RESULTS

The study included 98 patients diagnosed with CC with an average age of 49 years (77 women, 21 men).

**Figure 1:**
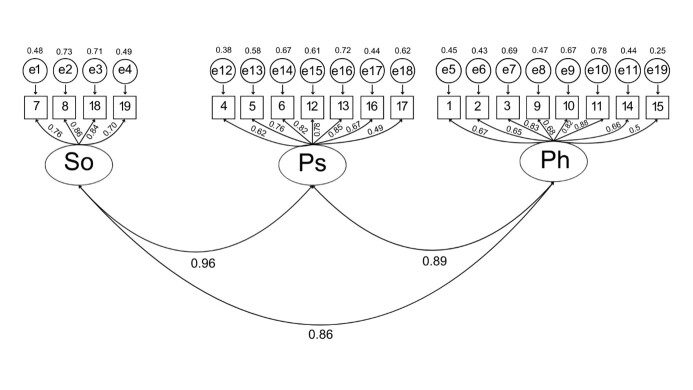
Factorial model of the Leicester Cough Questionnaire with three factors and 19 items

Confirmatory Factor Analysis (CFA) confirmed the factorial structure of the LCQ-Br, maintaining three factors and 19 items: physical (items 1, 2, 3, 9, 10, 11, 14, and 15), psychological (items 4, 5, 6, 12, 13, 16, and 17), and social (items 7, 8, 18, and 19), as shown in Figure 1. The adjustment indices were: 0.986 for GFI; 0.982 for AGFI; 0.773 for Parsimony Goodness-of-Fit Index (PGFI); 0.983 for NFI; 0.98 for RFI; 0.856 for PNFI; and 0.064 for SRMR, as shown in Table 1.

Caption: So = Social; Ps = Psychological; Ph = Physical

**Table 1:** Measures to adjust the factorial model of the Leicester Cough Questionnaire with three factors and 19 items

Model	GFI	AGFI	PGFI	NFI	RFI	PNFI	SRMR
Default model	0.986	0.982	0.773	0.983	0.980	0.856	0.064
Saturated model	1.000			1.000		0	
Independence model	0.171	0.079	0.154	0	0	0	
Zero model	0	0	0				

Confirmatory factor analysis, unweighted least squares

The internal consistency of the Leicester Cough Questionnaire was 0.889 for physical factors, 0.879 for psychological factors, 0.869 for social factors, and 0.952 for the complete instruments (Table 2).

**Table 2:** Analysis of the reliability by internal consistency of the Leicester Cough Questionnaire

	Cronbach’s alpha	Number of items
LCQ-Br Physical	0.889	8
LCQ-Br Psychological	0.879	7
LCQ-Br Social	0.869	4
LCQ-Br Total	0.952	19

Cronbach’s alpha

Table 3 shows that the reliability of the test-retest presented a coefficient value of 0.629 for the physical factor, 0.414 for the psychological, 0.332 for the social, and 0.455 for the total score.

**Table 3:** Reliability analysis by test and retest of the Leicester Cough Questionnaire

	ICC	CI 95%		F-test	df	^*^p-value
		Lower limit	Upper limit			
LCQ-Br Physical	0.629	0.230	0.821	2.692	30	0.004
LCQ-Br Psychological	0.414	-0.215	0.718	1.707	30	0.074
LCQ-Br Social	0.332	-0.385	0.678	1.497	30	0.137
LCQ-Br Total	0.455	-0.131	0.737	1.833	30	0.051

Intraclass correlation coefficient

Caption: df = degree of freedom; CI = confidence interval; F-test = measures the ratio of between-group variance to within-group variance;

* p-value = indicates statistical significance (p ≤ .05)

The concurrent validity showed a negative and significant correlation of the LCQ-Br physical, psychological, social and total factor, with the self-perception of laryngeal sensitivity (p<0.001; p=0.001; p=0.001; p<0.001, respectively), frequency (p<0.001; p<0.001; p<0.001; p<0.001; p<0.001, respectively) and intensity (p<0.001; p<0.001; p<0.001; p<0.001, respectively) of cough; with CSI-Br in the CSI-Br factors physical and social activities (p<0.001; p<0.001; p<0.001; p<0.001; p<0.001, respectively), CSI-Br Psychological and functional (p<0.001; p<0.001; p<0.001; p<0.001, respectively) and in the total score (p<0.001; p<0.001; p<0.001; p<0.001, respectively); and, positive correlation with the LHQ-Br (p<0.001; p<0.001; p<0.001; p<0.001, respectively), as shown in Table 4.

**Table 4:** Analysis of the criterion validity by concurrent validity of the Leicester Cough Questionnaire

		LCQ-Br	LCQ-Br	LCQ-Br	LCQ-Br
		Physical	Psychological	Social	Total
Self-perception sensitivity	r	-.441**	-.354**	-.348**	-.404**
	p-value	0.000	0.001	0.001	0.000
Frequency of cough	r	-.693**	-.623**	-.571**	-.676**
	p-value	0.000	0.000	0.000	0.000
Cough intensity	r	-.618**	-.630**	-.530**	-.640**
	p-value	0.000	0.000	0.000	0.000
Total CSI-Br	r	-.817**	-.751**	-.757**	-.827**
	p-value	0.000	0.000	0.000	0.000
CSI-Br Physical and social Activities	r	-.734**	-.683**	-.734**	-.756**
	p-value	0.000	0.000	0.000	0.000
CSI-Br Psychological and Functional	r	-.837**	-.748**	-.710**	-.821**
	p-value	0.000	0.000	0.000	0.000
LHQ-Br	r	.620**	.503**	.519**	.596**
	p-value	0.000	0.000	0.000	0.000

Spearman's correlation test

Values followed by ** indicate a p-value (probability value) ≤ 0.01, representing highly significant statistical significance. The p-value refers to the probability of obtaining extreme results under the null hypothesis, with low values (p ≤ 0.05) indicating evidence against this hypothesis

Caption: r = correlation coefficient

Table 5 shows that in responsiveness, there was a significant reduction in the Leicester Cough Questionnaire after the intervention in the physical (p=0.001), psychological (p=0.023), and total (p=0.009) factors.

**Table 5:** Analysis of the responsiveness of the Leicester Cough Questionnaire

Variable	Moment	Average	SD	Minimum	Maximum	1Q	Median	3Q	Z	^*^p-value
LCQ-Br Physical	Pre	5.19	1.70	1.00	7.00	3.81	5.38	6.94	-3.251	0.001
	Post	5.28	1.06	3.00	6.88	4.38	5.63	6.25		
LCQ-Br Psychological	Pre	4.61	1.63	0.88	6.13	3.38	5.38	6.13	-2.271	0.023
	Post	4.67	1.14	1.75	6.13	3.75	5.00	5.63		
LCQ-Br Social	Pre	5.72	1.74	1.00	7.00	4.63	6.75	7.00	-1.699	0.089
	Post	5.60	1.50	2.25	7.00	4.75	6.00	7.00		
LCQ-Br Total	Pre	15.52	4.73	3.13	20.13	12.25	17.25	19.81	-2.597	0.009
	Post	15.54	3.47	7.38	19.75	13.25	16.00	18.50		

Wilcoxon’s Test

* Values ≤ 0.05 indicate statistical significance; values ≤ 0.01 indicate high significance

Caption: SD = standard deviation; 1Q = first quartile; 3Q = third quartile; Z (Z-score) = Measure of how many standard deviations a result is above or below the population mean under the null hypothesis; p-value = Probability of obtaining results as or more extreme than those observed, assuming the null hypothesis is true

The final version of the LCQ is in Appendix B.

## DISCUSSION

The validation of the LCQ-Br in the Brazilian context marks an important advancement in the evaluation and follow-up of patients with CC. This instrument, initially developed in the United Kingdom, has proven to be a valuable tool for the evaluation of cough symptoms, offering a detailed perspective on the intensity and impact of cough on patients’ quality of life^(10,23)^.

The cultural adaptation of the LCQ-Br to BP and its validation are important steps to ensure the applicability and relevance of this questionnaire in a new demographic and cultural context. This validation process strengthens the evidence base for the clinical management of CC in Brazil and contributes to international knowledge about this prevalent condition, which interferes with the health and well-being of the patient^(9,23)^.

The results of the CFA adjustment indices indicate adequate adjustment of the model to the data. Specifically, indices such as GFI and AGFI close to 1 indicate adequate model fit, whereas a low SRMR value reinforces the adequacy of the data for the proposed model. Such metrics are essential for ensuring the accuracy and reliability of evaluation tools in clinical and research contexts^(24)^. These values suggest that the LCQ-Br is a reliable and valid instrument to assess the impact of CC in BP speakers.

Thus, in BP, the original factorial structure of the LCQ was maintained with three factors: physical, psychological, and social. Each factor had items that reflected the dimensions of the impact of CC on the individuals’ lives. This three-dimensionality favors an integral understanding of CC by associating it with the symptoms and psychological and social effects that often accompany this condition^(25,26)^.

The identification of the multifactorial nature of CC reaffirms the importance of multidimensional approaches in the treatment of CC. This highlights the need for interventions that are not limited to the relief of physical symptoms but address the psychological aspects that may be impaired in patients with CC^(27)^.

The availability of a validated instrument like the LCQ-Br facilitates the identification of specific areas of need for each patient, allowing for a personalized and effective approach to the management of CC. As CC is a complex condition, the integration of these aspects into patient care can significantly improve quality of life and treatment outcomes^(28)^.

The reliability of the instrument was verified through internal consistency, reliability tests, and retests. The results regarding the internal consistency of the LCQ-Br in the Brazilian context demonstrated its robustness and reliability as an evaluation tool. Values above 0.7 are expected, and those obtained in the instrument are higher than 0.85. The high internal consistency of the factors and the total score of the LCQ-Br reaffirm its usefulness in clinical and research contexts, offering a solid basis for future studies on CC and its management^(28)^.

Analysis of the reliability of the LCQ-Br test-retest suggested little stability of the responses over time. The expected values for this phase were above 0.7, and the physical domain approached this value, whereas the others decreased. The discrepancy in the values may indicate differences in the respondents' perceptions of the severity and impact of CC over time, especially in more subjective and variable aspects, such as emotional and social. Although the coefficient for the complete instrument is below the ideal value, it reflects the complexity and dynamic nature of CC, ratifying the importance of complementary approaches in the evaluation of this condition^(9,29)^.

Analysis of the concurrent validity of the LCQ-Br revealed negative and significant correlations between the scores of the physical, psychological, social, and total factors of the LCQ-Br and the measures of self-perception of laryngeal sensitivity, frequency, and intensity of the cough, as well as with the scores on the CSI-Br^(17)^, indicating a strong inverse association between the quality of life-related to cough and the perception of symptom severity.

A significant positive correlation was observed with the LHQ-Br^(16)^, reinforcing the relationship between the symptoms of laryngeal hypersensitivity and the perceived impact of cough. These results, consistent with the questionnaire domains and different aspects of cough experience, emphasize the ability of the LCQ-Br to comprehensively capture the impact of CC on the lives of individuals, evidencing its relevance and applicability for clinical evaluation and research in cough disorders. These significant negative and positive correlations not only demonstrate the concurrent validity of the LCQ-Br but also reinforce the importance of considering multiple dimensions of cough experience in the evaluation of the intensity and planning of the treatment^(29,30)^.

Analysis of the responsiveness of the LCQ-Br showed a significant decrease in scores after the intervention, covering the physical, and psychological domains in addition to the total score. These results indicate that this is a sensitive tool for detecting clinical changes in the construct after treatment. The ability of the LCQ-Br to capture these changes confirms its practical value as an evaluation tool, allowing health professionals to monitor the effectiveness of therapeutic interventions and adjust treatment strategies based on patient responses. Therefore, the significant reduction in scores reinforces the usefulness of the questionnaire not only for the initial evaluation of patients but also as a reliable indicator of clinical progress and improvement of cough-related quality of life^(27,28,30)^ (Appendix B).

## CONCLUSION

Validation of the LCQ-Br in the Brazilian context confirmed its position as an effective tool for diagnosing and monitoring CC. The LCQ-Br proved to be a valid and responsive instrument for obtaining information on the various effects of CC on patients' daily lives.

The questionnaire's responsiveness after therapeutic interventions emphasizes its clinical and research utility, allowing for effective monitoring of treatment outcomes and adaptation of CC management strategies. The implementation of the LCQ-Br as a standard evaluation tool for patients diagnosed with CC can facilitate a personalized treatment approach, contributing to an improved quality of life for patients.

Thus, the LCQ-Br is a valuable instrument for understanding and managing CC, supporting both clinical practice and research in this field.

## Appendix A Leicester Cough Questionnaire

**Table:**

Este questionário foi elaborado para avaliar o impacto da tosse em vários aspectos da sua vida. Leia cada pergunta com atenção e responda SELECIONANDO a resposta que melhor se aplica a você. Responda a TODAS as perguntas o mais honestamente possível.							
This questionnaire is designed to assess the impact of cough on various aspects of your life. Read each question carefully							
and answer by CIRCLING the response that best applies to you. Please answer ALL questions, as honestly as you can.							
1.	Nos últimos 14 dias, você teve dores no peito ou no abdômen por causa da tosse?						
1.	In the last 2 weeks, have you had chest or stomach pains as a result of your cough?						
							
	O tempo todo	Na maior parte do tempo	Uma boa parte do tempo	Uma parte do tempo	Uma pequena parte do tempo	Quase nunca	Nunca
	All of the time	Most of the time	A good bit of the time	Some of the time	A little of the time	Hardly any of the time	None of the time
2.	Nos últimos 14 dias, você ficou incomodado com a produção de catarro (muco) quando você tosse?						
2.	In the last 2 weeks, have you been bothered by sputum (phlegm) production when you cough?						
							
	Todas as vezes	A maioria das vezes	Várias vezes	Às vezes	Ocasionalmente	Raramente	Nunca
	Every time	Most times	Several times	Some times	Occasionally	Rarely	Never
3.	Nos últimos 14 dias, você ficou cansado por causa da tosse?						
3.	In the last 2 weeks, have you been tired because of your cough?						
							
	O tempo todo	Na maior parte do tempo	Uma boa parte do tempo	Uma parte do tempo	Uma pequena parte do tempo	Quase nunca	Nunca
	All of the time	Most of the time	A good bit of the time	Some of the time	A little of the time	Hardly any of the time	None of the time
4.	Nos últimos 14 dias, você sentiu que tinha controle da sua tosse?						
4.	In the last 2 weeks, have you felt in control of your cough?						
							
	Nunca	Quase nunca	Uma pequena parte do tempo	Uma parte do tempo	Uma boa parte do tempo	Na maior parte do tempo	O tempo todo
	None of the time	Hardly any of the time	A little of the time	Some of the time	A good bit of the time	Most of the time	All of the time
5.	Nos últimos 14 dias, você se sentiu envergonhado por causa da tosse?						
5.	How often during the last 2 weeks have you felt embarrassed by your coughing?						
							
	O tempo todo	Na maior parte do tempo	Uma boa parte do tempo	Uma parte do tempo	Uma pequena parte do tempo	Quase nunca	Nunca
	All of the time	Most of the time	A good bit of the time	Some of the time	A little of the time	Hardly any of the time	None of the time
6.	Nos últimos 14 dias, minha tosse me deixou ansioso						
6.	In the last 2 weeks, my cough has made me feel anxious						
							
	O tempo todo	Na maior parte do tempo	Uma boa parte do tempo	Uma parte do tempo	Uma pequena parte do tempo	Quase nunca	Nunca
	All of the time	Most of the time	A good bit of the time	Some of the time	A little of the time	Hardly any of the time	None of the time
7.	Nos últimos 14 dias, minha tosse afetou meu trabalho ou outras tarefas diárias						
7.	In the last 2 weeks, my cough has interfered with my job, or other daily tasks						
							
	O tempo todo	Na maior parte do tempo	Uma boa parte do tempo	Uma parte do tempo	Uma pequena parte do tempo	Quase nunca	Nunca
	All of the time	Most of the time	A good bit of the time	Some of the time	A little of the time	Hardly any of the time	None of the time
8.	Nos últimos 14 dias, senti que minha tosse não permitiu que eu aproveitasse minha vida no geral						
8.	In the last 2 weeks, I felt that my cough interfered with the overall enjoyment of my life						
							
	O tempo todo	Na maior parte do tempo	Uma boa parte do tempo	Uma parte do tempo	Uma pequena parte do tempo	Quase nunca	Nunca
	All of the time	Most of the time	A good bit of the time	Some of the time	A little of the time	Hardly any of the time	None of the time
9.	Nos últimos 14 dias, a exposição a tintas ou fumaças me fez tossir						
9.	In the last 2 weeks, exposure to paints or fumes has made me cough						
							
	O tempo todo	Na maior parte do tempo	Uma boa parte do tempo	Uma parte do tempo	Uma pequena parte do tempo	Quase nunca	Nunca
	All of the time	Most of the time	A good bit of the time	Some of the time	A little of the time	Hardly any of the time	None of the time
10.	Nos últimos 14 dias, sua tosse afetou seu sono?						
10.	In the last 2 weeks, has your cough disturbed your sleep?						
							
	O tempo todo	Na maior parte do tempo	Uma boa parte do tempo	Uma parte do tempo	Uma pequena parte do tempo	Quase nunca	Nunca
	All of the time	Most of the time	A good bit of the time	Some of the time	A little of the time	Hardly any of the time	None of the time
11.	Nos últimos 14 dias, quantas vezes por dia você teve ataques de tosse?						
11.	In the last 2 weeks, how many times a day have you had coughing bouts?						
							
	O tempo todo (continuamente)	A maioria das vezes ao longo do dia	Várias vezes ao longo do dia	Às vezes ao longo do dia	Ocasionalmente ao longo do dia	Raramente	Nenhuma vez
	All of the time (continuously)	2 Most times during the day	Several times during the day	Some times during the day	Occasionally through the day	Rarely	None
12.	Nos últimos 14 dias, minha tosse me deixou frustrado						
12.	In the last 2 weeks, my cough has made me feel frustrated						
							
	O tempo todo	Na maior parte do tempo	Uma boa parte do tempo	Uma parte do tempo	Uma pequena parte do tempo	Quase nunca	Nunca
	All of the time	Most of the time	A good bit of the time	Some of the time	A little of the time	Hardly any of the time	None of the time
13.	Nos últimos 14 dias, minha tosse me fez perder a paciência						
13.	In the last 2 weeks, my cough has made me feel fed up						
							
	O tempo todo	Na maior parte do tempo	Uma boa parte do tempo	Uma parte do tempo	Uma pequena parte do tempo	Quase nunca	Nunca
	All of the time	Most of the time	A good bit of the time	Some of the time	A little of the time	Hardly any of the time	None of the time
14.	Nos últimos 14 dias, você ficou com a voz rouca por causa da sua tosse?						
14.	In the last 2 weeks, have you suffered from a hoarse voice as a result of your cough?						
							
	O tempo todo	Na maior parte do tempo	Uma boa parte do tempo	Uma parte do tempo	Uma pequena parte do tempo	Quase nunca	Nunca
	All of the time	Most of the time	A good bit of the time	Some of the time	A little of the time	Hardly any of the time	None of the time
15.	Nos últimos 14 dias, você teve muita energia?						
15.	In the last 2 weeks, have you had a lot of energy?						
							
	Nunca	Quase nunca	Uma pequena parte do tempo	Uma parte do tempo	Uma boa parte do tempo	Na maior parte do tempo	O tempo todo
	None of the time	Hardly any of the time	A little of the time	Some of the time	A good bit of the time	Most of the time	All of the time
16.	Nos últimos 14 dias, você se preocupou com o fato de a tosse poder indicar uma doença séria?						
16.	In the last 2 weeks, have you worried that your cough may indicate serious illness?						
							
	O tempo todo	Na maior parte do tempo	Uma boa parte do tempo	Uma parte do tempo	Uma pequena parte do tempo	Quase nunca	Nunca
	All of the time	Most of the time	A good bit of the time	Some of the time	A little of the time	Hardly any of the time	None of the time
17.	Nos últimos 14 dias, você se preocupou que outras pessoas possam pensar que há algo errado com você por causa da tosse?						
17.	In the last 2 weeks, have you been concerned that other people think something is wrong with you, because of your cough?						
							
	O tempo todo	Na maior parte do tempo	Uma boa parte do tempo	Uma parte do tempo	Uma pequena parte do tempo	Quase nunca	Nunca
	All of the time	Most of the time	A good bit of the time	Some of the time	A little of the time	Hardly any of the time	None of the time
18.	Nos últimos 14 dias, minha tosse interrompeu conversas ou ligações telefônicas						
18.	In the last 2 weeks, my cough has interrupted conversation or telephone calls						
							
	Todas as vezes	A maioria das vezes	Uma boa parte do tempo	Uma parte do tempo	Uma pequena parte do tempo	Quase nunca	Nunca
	Every time	Most times	A good bit of the time	Some of the time	A little of the time	Hardly any of the time	None of the time
19.	Nos últimos 14 dias, senti que minha tosse incomodou meu cônjuge, minha família ou meus amigos						
19.	In the last 2 weeks, I feel that my cough has annoyed my partner, family or friends						
							
	Todas as vezes que tusso	A maioria das vezes quando tusso	Várias vezes quando tusso	Às vezes quando tusso	Ocasionalmente quando tusso	Raramente	Nunca
	Every time I cough	Most times when I cough	Several times when I cough	Sometimes when I cough	Occasionally when I cough	Rarely	Never
Obrigado por ter respondido a este questionário.							
Thank you for completing this questionnaire.							

## Appendix B Questionário de Leicester sobre Tosse (LCQ)

**Table:**

Este questionário foi elaborado para avaliar o impacto da tosse em vários aspectos da sua vida. Leia cada pergunta com atenção e responda SELECIONANDO a resposta que melhor se aplica a você. Responda a TODAS as perguntas o mais honestamente possível.							
1.	Nos últimos 14 dias, você teve dores no peito ou no abdômen por causa da tosse?						
							
	O tempo todo	Na maior parte do tempo	Uma boa parte do tempo	Uma parte do tempo	Uma pequena parte do tempo	Quase nunca	Nunca
2.	Nos últimos 14 dias, você ficou incomodado com a produção de catarro (muco) quando você tosse?						
							
	Todas as vezes	A maioria das vezes	Várias vezes	Às vezes	Ocasionalmente	Raramente	Nunca
3.	Nos últimos 14 dias, você ficou cansado por causa da tosse?						
							
	O tempo todo	Na maior parte do tempo	Uma boa parte do tempo	Uma parte do tempo	Uma pequena parte do tempo	Quase nunca	Nunca
4.	Nos últimos 14 dias, você sentiu que tinha controle da sua tosse?						
							
	Nunca	Quase nunca	Uma pequena parte do tempo	Uma parte do tempo	Uma boa parte do tempo	Na maior parte do tempo	O tempo todo
5.	Nos últimos 14 dias, você se sentiu envergonhado por causa da tosse?						
							
	O tempo todo	Na maior parte do tempo	Uma boa parte do tempo	Uma parte do tempo	Uma pequena parte do tempo	Quase nunca	Nunca
6.	Nos últimos 14 dias, minha tosse me deixou ansioso						
							
	O tempo todo	Na maior parte do tempo	Uma boa parte do tempo	Uma parte do tempo	Uma pequena parte do tempo	Quase nunca	Nunca
7.	Nos últimos 14 dias, minha tosse afetou meu trabalho ou outras tarefas diárias						
							
	O tempo todo	Na maior parte do tempo	Uma boa parte do tempo	Uma parte do tempo	Uma pequena parte do tempo	Quase nunca	Nunca
8.	Nos últimos 14 dias, senti que minha tosse não permitiu que eu aproveitasse minha vida no geral						
							
	O tempo todo	Na maior parte do tempo	Uma boa parte do tempo	Uma parte do tempo	Uma pequena parte do tempo	Quase nunca	Nunca
9.	Nos últimos 14 dias, a exposição a tintas ou fumaças me fez tossir						
							
	O tempo todo	Na maior parte do tempo	Uma boa parte do tempo	Uma parte do tempo	Uma pequena parte do tempo	Quase nunca	Nunca
10.	Nos últimos 14 dias, sua tosse afetou seu sono?						
							
	O tempo todo	Na maior parte do tempo	Uma boa parte do tempo	Uma parte do tempo	Uma pequena parte do tempo	Quase nunca	Nunca
11.	Nos últimos 14 dias, quantas vezes por dia você teve ataques de tosse?						
							
	O tempo todo (continuamente)	A maioria das vezes ao longo do dia	Várias vezes ao longo do dia	Às vezes ao longo do dia	Ocasionalmente ao longo do dia	Raramente	Nenhuma vez
12.	Nos últimos 14 dias, minha tosse me deixou frustrado						
							
	O tempo todo	Na maior parte do tempo	Uma boa parte do tempo	Uma parte do tempo	Uma pequena parte do tempo	Quase nunca	Nunca
13.	Nos últimos 14 dias, minha tosse me fez perder a paciência						
							
	O tempo todo	Na maior parte do tempo	Uma boa parte do tempo	Uma parte do tempo	Uma pequena parte do tempo	Quase nunca	Nunca
14.	Nos últimos 14 dias, você ficou com a voz rouca por causa da sua tosse?						
							
	O tempo todo	Na maior parte do tempo	Uma boa parte do tempo	Uma parte do tempo	Uma pequena parte do tempo	Quase nunca	Nunca
15.	Nos últimos 14 dias, você teve muita energia?						
							
	Nunca	Quase nunca	Uma pequena parte do tempo	Uma parte do tempo	Uma boa parte do tempo	Na maior parte do tempo	O tempo todo
16.	Nos últimos 14 dias, você se preocupou com o fato de a tosse poder indicar uma doença séria?						
							
	O tempo todo	Na maior parte do tempo	Uma boa parte do tempo	Uma parte do tempo	Uma pequena parte do tempo	Quase nunca	Nunca
17.	Nos últimos 14 dias, você se preocupou que outras pessoas possam pensar que há algo errado com você por causa da tosse?						
							
	O tempo todo	Na maior parte do tempo	Uma boa parte do tempo	Uma parte do tempo	Uma pequena parte do tempo	Quase nunca	Nunca
18.	Nos últimos 14 dias, minha tosse interrompeu conversas ou ligações telefônicas						
							
	Todas as vezes	A maioria das vezes	Uma boa parte do tempo	Uma parte do tempo	Uma pequena parte do tempo	Quase nunca	Nunca
19.	Nos últimos 14 dias, senti que minha tosse incomodou meu cônjuge, minha família ou meus amigos						
							
	Todas as vezes que tusso	A maioria das vezes quando tusso	Várias vezes quando tusso	Às vezes quando tusso	Ocasionalmente quando tusso	Raramente	Nunca
Obrigado por ter respondido a este questionário.							
